# Successful population establishment from small introductions appears to be less common than believed

**DOI:** 10.7717/peerj.2440

**Published:** 2016-09-21

**Authors:** Alyssa Corbett King, J. Michael Reed

**Affiliations:** Department of Biology, Tufts University, Medford, MA, USA; 2 Current affiliation: The School for Field Studies, Beverly, MA, USA

**Keywords:** Population viability, Translocation, Small population paradigm, Propagule pressure, Invasive species, Allee effect, Introduction success

## Abstract

Although small populations are at high risk of extinction, there are regular reports in the scientific literature of purported small, isolated, persistent populations. One source of evidence of the viability of small populations comes from the alleged successful introduction of species to areas outside their original range from introductions of few individuals. We reviewed the examples from introduction compendia on deliberate translocations of birds, and the original sources, to identify and evaluate purported examples of successful establishments from small introductions. We found 23 purportedly successful introductions from few (<30) individuals. After assessing original sources, we found that two of the claims were substantiated; the rest were ambiguous or could be rejected as examples, primarily due to a lack of evidence in original sources of the number of birds released and because of supplemental individuals from other releases, releases in nearby regions, and the possibility of natural invasion. Our results suggest that reports of successful establishment of birds from introductions of few individuals have been overstated. These results strengthen the relationship previously reported between propagule pressure and likelihood of establishment, and support the lack of viability of small populations presumed by population theory. We suggest that analyses of introduction failure and success would benefit from excluding studies where introduction effort is unknown or unreliably documented.

## Introduction

Despite wide acknowledgement that small populations are at greater risk of extinction than are larger populations (e.g., MacArthur & Wilson, 1967; Gilpin & Soulé, 1986; Ludwig, 1996), there are persistent caveats in the scientific literature that small, isolated populations can persist (e.g., Simberloff, 1998; Walters & Crist, 2006). The highest profile recent example is that of the purported rediscovery of the Ivory-billed Woodpecker (*Campephilus principalis*) (Fitzpatrick et al., 2005, but see Sibley et al., 2006; Scott et al., 2008; Elphick, Roberts & Reed, 2010). These examples appear to defy the small-population paradigm, described as the tendency for small populations to succumb to stochastic events and extinction vortices (Gilpin & Soulé, 1986; Caughley, 1994; Fagan & Holmes, 2006; Brook, Sodhi & Bradshaw, 2008). There are few examples of persistent, small, isolated populations of vertebrates, and possibly none that have been investigated in detail. For example, although the Devil’s Hole Pupfish (*Cyprionodon diabolis*) is endemic to a single pool in the Mojave Desert and the pond has been isolated for at least 60,000 years (Riggs & Deacon, 2004), it appears that the fish have not been there for more than a few thousand years, and possibly much less time (Reed & Stockwell, 2014; Martin et al., 2016; but see Sağlam et al., 2016 for a contrary assessment). One source of evidence related to the potential viability of small populations comes from species introductions to new regions (Duncan, Blackburn & Sol, 2003; Blackburn, Lockwood & Cassey, 2009; Blackburn et al., 2011). Evaluating the success or failure of deliberate species introductions has provided important insight into population dynamics and invasion ecology.

Avian introductions have been of particular interest because of the extensive number of species introductions during the 19th and 20th centuries during and after colonial European expansion. The most distinctive pattern that has emerged from the analyses of bird introductions is that introduction effort, in terms of the number of individuals per release and number of releases (sometimes referred to as ‘propagule pressure’, e.g., Simberloff, 2009), is an important factor determining successful establishment (Griffith et al., 1989; Wolf et al., 1996; Green, 1997; Sol & Lefebvre, 2000; Brook, 2004; Lockwood, Cassey & Blackburn, 2005; Blackburn, Lockwood & Cassey, 2009; Blackburn et al., 2011; Blackburn et al., 2013; Hufbauer et al., 2013; but see Moulton, Cropper & Avery, 2012). Despite the common outcome that introductions of few individuals (low propagule pressure) tend to fail, there are regular reports of successful establishment from the introduction of very few individuals (Newsome & Noble, 1986; Tomich, 1986; Pimm, 1989; Pimm et al., 1993; Green, 1997; Simberloff, 1998; Taylor, Jamieson & Armstrong, 2005). The idea that successful introduction can occur from the introduction of few individuals has been called the Noah fallacy (Simberloff, 2009).

In impressive compendia, Long (1981), Lever (1987) and Lever (2005) reviewed successful and failed attempts at bird species introductions, and they regularly report successful introductions from few individuals. In fact, Long (1981) is the primary source for most published studies that evaluate patterns of extinction risk. Our goal was to assess the quality of the evidence cited to support successful establishment from introductions of very few individuals. We did this specifically for birds because this taxon has the most extensive available data, and these data are regularly cited as providing examples of successful introductions. Previous studies have shown that invasion success by introduced birds is correlated with a variety of factors, including introduction effort (e.g., Griffith et al., 1989; Wolf et al., 1996; Sol & Lefebvre, 2000; Lockwood, Cassey & Blackburn, 2005). In addition, it is generally viewed that introductions of fewer than 5 individuals is not likely to lead to success (Moulton & Pimm, 1983; Cassey, 2002). However, assessments regularly identify apparent exceptions to the rule—purported introductions of few birds that somehow establish. One recent example is by Moulton et al. (2010), who argue that there is no real evidence that House Sparrow (*Passer domesticus*) establishment in North America came from more than the original release of 16 individuals, rather than from multiple introductions of hundreds of birds, as is traditionally argued (Long, 1981). Duncan (1997, p. 905), for example, states that there are “recorded instances of successful invasion following the release of only two birds (Drummond, 1906).” However, if one reads the original source for this example, Drummond (1906, p. 243) states: “Many years ago a pair of these birds… nested in a kauri-tree about a hundred yards from a settler’s house, and from that spot they completely spread throughout the whole country.” So for this example there is no indication of the number of individuals originally introduced to the area, and the referenced pair could have been one of hundreds introduced to the region. Consequently, it would not be valid to assume that the existing population did indeed arise from a single introduction of two individuals.

Moulton et al. (2010) suggest that historical records need to be critically evaluated. Many of the examples reported of introduction success of birds from few individuals come from summary reports rather than from the original records. Here we evaluate alleged examples of introduction success of bird species from low introduction effort (low propagule pressure) from large summary reports (Long, 1981; Lever, 1987; Ebenhard, 1988) by examining original sources of these examples to determine the level of support that these were isolated introductions of the stated small size. This is not intended to be a review of all reported examples; it is an assessment of examples from the most commonly cited compendia. If we find any examples of understated introduction effort, or insufficiently documented support of introduction numbers, we will support our premise that successful introduction from few individuals is unlikely, or at least less likely than currently presumed.

## Methods

We gathered assertions of persistent populations arising from non-native introductions of few individuals reported in compendium volumes by Long (1981), Lever (1987), and Ebenhard (1988); specific references were not available for some compendia (e.g., Newsome & Noble, 1986; they report introduction successes from few individuals but do not include references, citing a library repository that no longer has them). It was not our goal to do a thorough literature review or all alleged examples because it turned out to be unnecessary for our assessment, and the summary reports are the sources of most cross-species analyses. We considered an introduction to be “few” individuals if it was of 30 or fewer birds; this population size is easily small enough that extinction due to demographic and environmental stochasticity is expected to drive a species extinct, yet there are published assertions of successful establishment from these numbers. We ignore the lack of a standard definition of success, and assume that a population currently extant represents a successful introduction. We traced each cited example back to its original sources and scored the assertions for two measures: (1) confidence of the number released, and (2) confidence that there were no subsequent releases, no other nearby releases, and no likelihood of natural invasion. The confidence of number released was scored as: 0 = no evidence of actual number released; 1 = numbers reported, but no indication that it was of the complete introduction; 2 = second hand report of carefully documented release; 3 = quantitative documentation by person doing the release, and no other documentation of releases. The confidence that there was only one, isolated release was scored as: 0 = known other releases; 1 = suspected other releases; 3 = specifically addressed in literature that no other releases occurred. (There was no similar intermediate value for a confidence score of 2; we skipped it so that 3 would represent a similar level of confidence.) We also included relevant information from other sources not cited by the analysis papers.

Because it is difficult to assess whether or not an introduced population remained isolated during colonization, we recorded documented instances of nearby introductions as possible sources of population augmentation. We defined “nearby” as any introduction that is within a plausible dispersal or individual exploration distance. For example, most of the New Zealand introductions have been considered as isolated in the specific province in which the release occurred. However, there is evidence of the song thrush (scientific names are found in Table 1) dispersing between provinces, suggesting that it is likely that other introduced populations were able to disperse in a like manner (Lever, 1987, p. 333). Similarly, Long (1981, p. 38) reported that black swans appeared hundreds of kilometers away from many of their release points in New Zealand (scientific names are found in Table 1), allowing for the complete colonization of the country and other offshore islands. Therefore, it is logical to include nearby releases as possibly augmenting the original founder population.

**Table 1:** Evaluation of purported successful establishments of species from single introductions of few (<30) individuals; details from primary and secondary sources in Supplemental Information.

Species	Number reported released	Where introduced	Cited by	Confidence in		Rationale for confidence scores based on primary and secondary sources	Assertion accepted?
				Number released^a^	Lack of supplemental individuals^b^		
Mute swan *Cygnus olor*	12	Australia	Duncan et al. (2001)	0	0	Uncertainty about accuracy of introduction data; likely previously established wild population	No
	29	New Zealand	Veltman, Nee & Crawley (1996)	0	0	Additional birds released; possibility of earlier introductions	No
Black swan *C. atratus*	4	Auckland, NZ	Lever (1987)	2	0	No first-hand documentation of release; multiple releases to nearby regions	No
Cape Barren goose *Cereopsis novaehollandiae*	8^c^	New Zealand	Veltman, Nee & Crawley (1996)	1	0	Subsequent introductions; no first-hand documentation of release; possibility of natural invasion	No
Cattle egret *Bubulcus ibis*	18	Australia	Duncan et al. (2001)	2	0	Introduced birds apparently disappeared; no first-hand documentation of release; possible establishment via natural invasion	No
	12–21	Chagos Archipelago	Lever (1987)	1	–	Uncertainty about number released; possibility of other releases not addressed	No
Laughing dove (Senegal turtledove) *Streptopelia senegalensis*	4	Australia	Duncan et al. (2001)	0	–	No data available on number introduced; possibility of other releases not addressed	No
Laughing kookaburra *Dacelo novaeguineae*	21	New Zealand	Veltman, Nee & Crawley (1996)	0	–	No data available on number introduced; possibility of other releases not addressed	No
Australian magpie *Gymnorhina tibicen*	2	Australia	Duncan (1997)	0	–	No data available on number introduced; possibility of other releases not addressed	No
	10	Aukland, NZ	Duncan (1997)	2	0	No first-hand documentation of release; may have previously been present in the wild; multiple releases at nearby sites	No
Song thrush *Turdus philomelos*	8	Wellington, NZ	Duncan (1997)	2	0	No first-hand documentation of release; multiple releases at nearby sites	No
Red-whiskered bulbul *Pynconotus jocosus*	8	Australia	Duncan et al. (2001)	0	–	No data available on number introduced; possibility of other releases not addressed	No
	10–20	Miami, USA	Lever (1987)	0	–	No data available on number introduced; possibility of other releases not addressed	No
Eurasian tree sparrow *Passer montanus*	20–24	St. Louis, USA	Lever (2005)	2	3	Supported	Yes
House sparrow *P. domesticus*	14^d^	Otago, NZ	Duncan (1997)	2	0	No first-hand documentation of release; 2 releases at introduction site; multiple releases at nearby sites	No
Scaly-breasted munia (spice finch) *Lonchura punctulata*	8	Australia	Duncan et al. (2001)	0	–	No data on number introduced; possibility of additional releases not addressed	No
Chaffinch *Fringilla coelebs*	16	Canterbury, NZ	Duncan (1997)	0	0	Additional birds were released at same site; multiple releases at nearby regions	No
Island canary *Serinus canaria*	12^e^	Midway Atoll, Hawaii	Lever (1987)	3	3	Supported	Yes
European greenfinch *Carduelis chloris*	8	Otago, NZ	Duncan (1997)	1	0	No first-hand documentation of release; use of the same common name for different species; multiple releases at nearby sites	No
Common redpoll *Carduelis flammea*	2	Wellington, NZ	Duncan (1997)	2	0	No first-hand documentation of release; multiple releases at nearby sites	No
Cirl bunting *Emberiza cirlus*	29^f^	New Zealand	Veltman, Nee & Crawley (1996)	1	0	For all 3 cases: no first-hand documentation of releases; multiple releases at nearby sites; other suspected introductions	No
	7	Otago, NZ	Duncan (1997)	2			
	4	Wellington, NZ	Duncan (1997)	2			

**Notes.**

a
0 no evidence of actual number released 1 numbers reported, but no indication that it was of the complete introduction 2 second hand report of carefully documented release 3 quantitative documentation by person doing the release

b Via subsequent releases, other nearby releases, or natural invasion: 0, known other releases; 1, suspected other releases; 3, specifically addressed in literature that no other releases occurred; dash, not addressed by primary sources.

c Original records indicate 6 (Williams, 1968:67).

d Duncan (1997) cites Long (1981), who says 23 were released, but Long cites Thomson (1922) who says 14 were released.

e Original records indicate 13 (Bryan, 1912).

f Original records indicate 11, as 18 released on an island failed (Thomson, 1922:175, Long, 1981:466).

## Results

**Figure 1:**
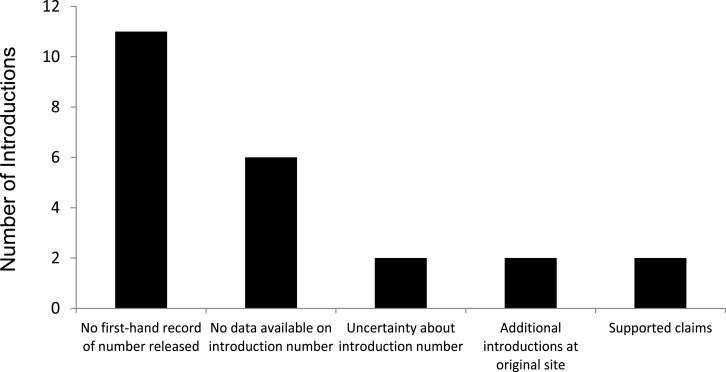
Assessments of 23 successful introductions of 17 species of birds alleged to have established from a single, isolated introduction of few (<30) individuals.

We evaluated 23 introductions that included 17 species, and recorded the confidence levels for each introduction (Table 1); details of what was reported in the original citations are found in Supplemental Information. Of the 23 introductions, we found only two that were strongly supported by original sources. One of the species reported as a successful introduction, the Cape Barren goose to New Zealand, has become extirpated so it cannot be considered a translocation success (Lever, 1987; Falla, Sibson & Turbott, 1966). Lack of support, or ambiguous support, for the reported number being released fell into four categories, including no first-hand documentation of release, no data available on introduction number, uncertainty about data available on introduction number, and additional releases at or near the original release site (Fig. 1).

**Figure 2:**
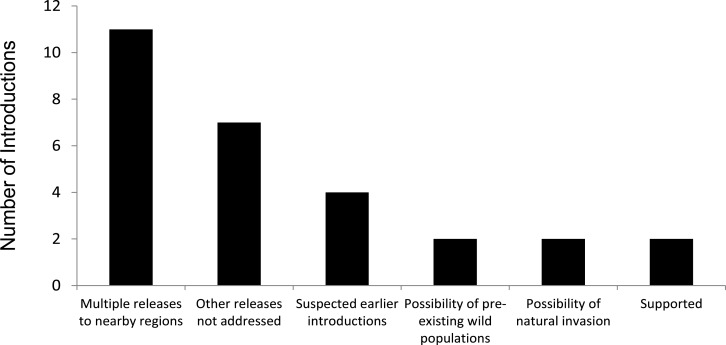
Assessment of support for releases being a single, isolated event for 23 successful introductions of 17 bird species alleged to have established from a single, isolated introduction of <30 individuals. (Note: ‘Possibility of natural invasion’ refers to species where there is the suggestion that the species’ presence might have included unaided invasion.)

Similarly, for 21 of the 23 introductions we had low confidence that the established population came from the introduction specified by the analytical review papers and compendium volumes. We found that the original literature for introductions report known additional introductions in the region, suspected additional introductions, had a lack of information addressing whether or not there were other releases in the general region as the original release in the same time period, acknowledged possibilities that a wild population of the species was already present in the same region, or that the current population might have come from a natural invasion (Fig. 2).

Finally, there can be complications from use of non-standard or inaccurate bird names that can cause both types of error. Williams (1968), for example, reported that the European greenfinch was sometimes referred to as the green linnet, thus making it impossible to determine the exact number of birds released for some species.

## Discussion

Although we found reports of 23 successful introductions from a total of 17 species of birds that were purported to have included fewer than 30 individuals in the introduction, only two appear to be strongly supported by data. The first is the island canary, which was introduced to the Hawaiian island of Midway in July of 1910. Only 11–14 canaries were released, but it was estimated that about 60 young were raised in the wild during the first breeding season, and the species is now common on the island (Bryan, 1912; Munro, 1944; Berger, 1981). The introduction, however, was facilitated by extensive human activities. Cats were removed from the island before birds were released, and released canaries were supplied for years with food from feeders (Munro, 1944; Berger, 1981). Also, a couple living on the island kept caged canaries for years (Munro, 1944), so it is not impossible that there were additional releases or escapes. The other example of an apparently successful release from few individuals is the Eurasian tree sparrow in St. Louis (USA), which apparently originated from an introduced population of 20–24 birds released in 1870 (Phillips, 1928; Wetmore, 1964; Barlow, 1973). We were not able to find evidence of additional releases (Cooke & Knappen, 1941).

In contrast, 21 of the reported successful introductions from a small number of individuals were either not supported by original sources, or the information was ambiguous. The primary reason for rejecting the validity of a purported successful introduction from a small release group was a lack of evidence for the number of birds actually released. A particularly interesting example comes from the introduction of the red-whiskered bulbul to Florida (USA). The claim is that the initial introduction was of 10–20 birds, but this appears to be a guess based on possible population growth (Carleton & Owre, 1975, pp. 43–44): “To produce the present population, a founding population of fewer than five feeding pairs would have had an annual rate of increase of more than 50%—which seems excessive. An initial population of more than 10 pairs seems too large from accounts of residents…Thus, from tentative data, we hypothesize that between 5 and 10 breeding pairs founding the population…” This is an excellent example of how speculation can transform over retelling into apparent fact.

Another common error is that reports ignored multiple additional introductions of the same species to nearby regions before successful establishment was documented. An example of this is the Australian magpie introduction to Auckland, New Zealand. The implication in the analytical literature is that the 1867 introduction of 10 birds (and one more in 1870) was an isolated event that led to establishment of the Australian magpie in the province (Duncan, 1997). However, original sources show that there were at least 6 other releases to New Zealand within a decade of the original release (Anderson, 1916; Thomson, 1922; Lamb, 1964; Wellwood, 1968; Williams, 1969). The entire suite of releases might then form the basis for the current population, although without data that are apparently unavailable we cannot determine the functional size of the starting population.

Our results strengthen the importance of the observed relationship between propagule pressure to introduction success by showing that many apparent exceptions are, or might be, invalid. This supports the conclusions from earlier analyses, and is in contrast to results reported by Nuñez, Moretti & Simberloff (2011), who reported plant invasion to be unrelated to the number of individuals introduced. Our analysis indicates that in fact 21 of those are not supported or have insufficient data to be evaluated, leaving only two apparently valid examples. These successes might be viewed as unusual chance events that are expected from a large number of attempts with a low likelihood of success, or as examples where there were additional releases not recorded any place we have found, or that records of actual additional releases were not kept. Moulton et al. (2010) provide another example of this regarding the history of introductions of house sparrows to North America, with assessments ranging from 8 pairs to over 50, with the actually number of birds released not determinable from the conflicting historic records.

We are not stating that successful introductions from few individuals are not possible, and they continue to be asserted (e.g., Van Houtan et al., 2009). Rather, that it is a statistical unlikelihood, so that claims of this type of success should be evaluated critically. One possibility that has not been raised in the small-population literature is that the dynamics of introduced populations are fundamentally different than those of naturally small or artificially reduced populations. This might make an interesting avenue for future investigation. In addition, it would be valuable to decide on unambiguous criteria for defining of success of introduction that distinguishes establishment from invasion and includes a time frame. For example, the crested myna *Acridotheres cristatellus* was introduced to Vancouver, British Columbia, Canada in the late 1890s. The species became established, invaded, increasing in numbers to a peak of around 20,000 birds in the late 1920s, and then decreased to extinction in 2003 (Long, 1981; Simberloff & Gibbons, 2004). The mynas certainly successfully established, as can occur from introductions of few individuals (e.g., Szűcs et al., 2014), but does this count as an invasion success because it became abundant and persisted over 100 years, or as a failure because failed to persist much longer? Consistent with our message in this manuscript of the importance of reading original sources, Long (1981) reports that this species was “certainly known” to be in British Columbia in 1894, citing Wood (1924) for the information. This assertion has been reprinted subsequently by later sources, e.g., Lever (2005). However, reading the original source, Wood states that the species was “certainly unknown” in British Columbia in 1894 (emphasis ours).

Our results also suggest that analyses of invasion success should include a critical evaluation of the evidence for success and propagule size (also argued by Moulton et al., 2010; Moulton, Cropper & Avery, 2011; Blackburn et al., 2013). Studies that analyze introduction data are sometimes careful in their methods to state that the number of animals introduced that is analyzed is the *minimum* number released, but the results and interpretation often subsequently treat this value as the actual number released, which introduces a source of error into the statistical analyses and interpretations. What might be considered a gold standard is to only include species where the historical record is extensive—including number of animals released and a discussion that includes reference to a lack of other releases. Regardless, historic records will often be incomplete. In the absence of sufficiently robust records, one has few choices: (1) do not do the analysis, (2) do a qualitative analysis, (3) do the analysis, but carefully couch the conclusions in the context of perceived data quality, and perhaps (4) include sensitivity analyses of the effects of different data values on the results.

##  Supplemental Information

10.7717/peerj.2440/supp-1 Supplemental Information 1 Information from primary sources Quotes from the original literature relating to the confidence scores in Table 1; information from the first 3 columns are found in Table 1. “Analysis Paper” refers to the analytical paper evaluating factors related to introduction success; “Secondary Citation Source” refers to the source cited by the Analytical Paper, and the “Secondary Documentation” quotes the relevant part of the Secondary Citation Source. The “Original Citation” was cited by the Secondary Source, and the “Original Documentation” quotes the relevant part of the Original Citation. “Notes” refers to sources of uncertainty, which affected the confidence scores in Table 1. Purported successful introductions that did not come from the Analysis Papers. We also include what we view as other relevant sources of information, and they are labeled under Analysis Paper as “Information from other literature”, or as a “Secondary Citation Source” but with no associated Analysis Paper. The final column lists other sources listed by the analysis paper for that species that we checked and found that they did not contain data on introduction numbers.

## Additional Information and Declarations

### Data Availability

The following information was supplied regarding data availability: The raw data has been supplied as Supplemental Information.
