# Supplementary material for: Successful population establishment from small introductions appears to be less common than believed
Source: PeerJ. 2016 Sep 21;4:e2440. doi: 10.7717/peerj.2440 (PMC5036100; doi:10.7717/peerj.2440)
Supplement: Supplemental Information 1 — Quotes from the original literature relating to the confidence scores in Table 1; information from the first 3 columns are found in Table 1. “Analysis Paper” refers to the analytical paper evaluating factors related to introduction success; “Secondary Citation Source” refers to the source cited by the Analytical Paper, and the “Secondary Documentation” quotes the relevant part of the Secondary Citation Source. The “Original Citation” was cited by the Secondary Source, and the “Original Documentation” quotes the relevant part of the Original Citation. “Notes” refers to sources of uncertainty, which affected the confidence scores in Table 1. Purported successful introductions that did not come from the Analysis Papers. We also include what we view as other relevant sources of information, and they are labeled under Analysis Paper as “Information from other literature”, or as a “Secondary Citation Source” but with no associated Analysis Paper. The final column lists other sources listed by the analysis paper for that species that we checked and found that they did not contain data on introduction numbers. [file peerj-04-2440-s001.pdf]

Supplemental material

Quotes from the original literature relating to the confidence scores in Table 1; information from the first 3 columns are found in Table 1. For cited books, the citation is given as year:page number. "Analysis Paper" refers to the analytical paper evaluating factors related to introduction success; "Secondary Citation Source" refers to the source cited by the Analytical Paper, and the "Secondary Documentation" quotes the relevant part of the Secondary Citation Source. The "Original Citation" was cited by the Secondary Source, and the "Original Documentation" quotes the relevant part of the Original Citation. "Notes" refers to sources of uncertainty, which affected the confidence scores in Table 1. Purported successful introductions that did not come from the Analysis Papers. We also include what we view as other relevant sources of information, and they are labeled under Analysis Paper as "Information from other literature", or as a "Secondary Citation Source" but with no associated Analysis Paper. The final column lists other sources listed by the analysis paper for that species that we checked and found that they did not contain data on introduction numbers.

**Common Name** ***Scientific Name***	**Asserted number of individuals released (location)**	**Analysis paper**	**Secondary citation source**	**Secondary documentation**	**Original citation**	**Original documentation**	**Notes**	**Other citations checked**
Mute swan *Cygnus olor*	12 (Australia)	Duncan *et al.* 2001	Long 1981:38	"A pair of white swans were released on Phillip Island, Victoria, in 1866 (Jenkins 1977, Balmford 1978) and four white swans arrived in Melbourne… Both imports were probably mute swans. Balmford also records that a single white swan may have been noted in the wild before 1859…Six birds were released in Launceston, Tasmania in the 1920s and progeny from these were released in other areas (Jenkins 1977)."	Jenkins 1977:24, 76	24: "Highly prized arrivals were three pairs of white (mute) swans from the Vintners Company of London which arrived on S.S. *Devon* in 1897. Two pairs were allocated to the City of Perth and one pair to the zoo. Later records in 1912 state swans were 'turned out for acclimatization' and the breeding colony on the Avon River at Northam may have come from this source."<br><br>76: Reports two white swans were released on Phillip Island in 1866 and others were released in various Town Councils. Reports the population did not establish.	Uncertain data: "Both imports were probably mute swans" (Long 1981:38).<br><br>Birds may have been present in the wild before introduction (Balmford 1978:244, Lever 1987:27).<br><br>The two swans released to Phillip Island in 1866 were said to not have established (Jenkins 1977:76).<br><br>Numbers of birds introduced differs between references.	Balmford 1978:240

			Newsome and Noble 1986:17	Appendix 1 Approximate number of individuals released: “?>20”	Sources not cited, and are unavailable where the paper says they are stored			
		Information from other literature:	Lever 1987:27	“Semi-wild populations have occurred since before 1912…”				
	29 (New Zealand)	Veltman *et al.* 1996	Long 1981:37	“Six Mute Swans were introduced at Canterbury in 1966, nine at Otago in 1968-69, fourteen at Auckland in 1969-71, and several birds were introduced by private individuals and dealers at about the same time (Thomson 1922). Other earlier introductions include some which were made at Christchurch 1866, Auckland in 1867, and Dunedin in 1868, when two or more birds were released at a time (Oliver 1955)”.	Thomson 1922:107	“The Canterbury Society received two in 1966; the Otago Society three in 1968, and one in 1989; the Auckland Society two in 1869 (from Sire Geo. Grey), and 12 in 1871 (from captain Hutton). Several were also introduced by private individuals and by dealers. It is nowhere common.”	Population may have been increased by the release of several birds by private individuals (Thomson 1922:107) “about the same time,” (Long 1981:37).<br><br>Earlier introductions occurred, indicating the possibility for existing populations at the time of the cited release (Oliver 1955).	Cramp 1997:374

Black swan *Cygnus atratus*	4 (Auckland)		Lever 1987:30	"According to Buller (1872), Black Swans were first introduced into the North Island in 1864. The earliest introduction to that island traced by Thomson, however, was in 1867, when the Auckland Acclimatization Society released four."	Thomson 1922:107	"The Auckland Society liberated four in 1867."	Multiple other releases occurred in nearby locations in the same decade, including Canterbury (11-20 ind., 1864-1866, Thomson 1922:107, Lever 1987:30), Nelson (7 ind., 1864, Long 1981:38) and Otago (61 ind., 1866-1870, Thomson 1922:107), and Southland (6 ind., 1869, Thomson 1922:107).	Buller 1872
Cape barren goose *Cereopsis novaehollandiae*	8 (New Zealand)	Veltman *et al* 1996	Long 1981:40	"…[this] introduction may have been temporarily successful. In 1912, the Otago Society received two Cape Barren Geese and bred them at the Government Poultry Farm at Milton. Four of the progeny were liberated at Lake Hawea in 1914 or 1915, and the remaining birds were sent to a hatchery at Clinton (Thomson	Thomson 1922:106	"In 1912 the Otago Society received two, and sent them to the Government Poultry Farm at Milton, where several young were reared. From these, four were placed at the head of Lake Hawera in 1915, and others sent down to the Society's hatchery at Clinton. They appear to be doing well by latest reports."	Two releases occurred in same district (Otago), including 4 in 1914/1915 and 2 in 1917 (Thomson 1922:106).<br><br>Birds may have dispersed to New Zealand naturally, and not the result of a human introduction (Falla *et al.* 1966:67, Williams	

				1922, Williams 1968). According to Williams, five young were reared on Lake Hawea in 1916 and more the following year. A pair of birds from Clinton was liberated at Minarets Station, Lake Wanaka in 1917."<br><br>"Wodzicki (1965) records that the species is still established locally in the South Island of New Zealand, but Falla *et al.* (1966) indicate that there are no subsequent records after 1947. It is doubtful that the species is at present established in New Zealand."	Williams 1968:67	"Importations were made to New Zealand in 1869, 1871, and 1912 (Thomson 1922). The first two came to nothing, but the third, made by the Otago Acclimatisations Society – was at least temporarily successful: The single pair bred at the Government Poultry Farm at Milton and some of the offspring were sent to the Society's aviary at Clinton where they too, bred, after reaching the age of three years. In 1914 four were liberated at the head of Lake Hawea and five young were reared there in 1916. A year later the birds apparently bred again at Lake Hawea and a pair from Clinton was liberated at Minarets Station, Lake Wanaka. The Annual Reports of the Otago Acclimitisation	1968:87).<br><br>It is likely that the goose is not established in New Zealand (Falla *et al.* 1966:87, Lever 1987:38)	

						Society (from which these details have been obtained) contain no other explicit references to the birds breeding in the wild… Apparently birds not liberated in previous years or kept for breeding were given to private persons and their fate is unknown.”<br><br>“Other reports of the species in New Zealand apparently refer to birds carried over 1300 miles from Australia by the prevailing westerly or north-westerly weather systems (as have other Australian species from time to time – Williams, 1963, p. 56). However, the birds concerned may have been strays or descendents from the Wanaka-Hawea population.”		

Cattle egret *Bubulcus ibis*	18 (Australia)	Duncan *et al.* 2001	Long 1981:71	"Colonisation of Australia by the Cattle Egret appears to have begun in the 1940s. They were first noted in the Oenpelli area, Arnhem Land, (Chalmers 1972) in the Northern Territory in 1948 (Morgan and Morgan 1965), and subsequently in many parts of the eastern States and Western Australia. Accounts of their spread in Australia are given by Hewitt (1960), Jenkins and Ford (1960), Wheeler (1962), and others."	Chalmers 1972:180<br><br>Hewitt 1960:99	"…were introduced into Australia in 1933 in the Kimberley region when 18 were released there, though apparently they did not become established (Serventy and Whittell 1948:170).<br><br>"The existence of a large population of Cattle Egrets in Northern Australia, and the presence of the species in other widely-separated areas in Australia, suggests a reconsideration of the theory sometimes advanced that the Cattle Egret in Australia is descended from some 18 birds imported from India and liberated near Derby, Western Australia, in 1933 (Serventy and Whittell 1948:170)…In 1948, H.G. Deignana of the National Geographic-Smithsonian	It is possible that the Australian population of cattle egret could have been due to natural dispersal, rather than introduced by man (Hewitt 1960:99, Newsome and Noble 1986:17, Lever 1987:17).<br><br>The 18 birds released are recorded by multiple sources to have not established (Serventy and Whittell 1948:170, Lever 1987:17).	Jenkins and Ford 1960:245-249, Wheeler 1962:192, Morgan and Morgan 1965:230

						Institution Arnhem land Expedition, observed ‘hundreds’ of Cattle Egrets in the Oenpelli district of the Northern Territory. He did not subscribe to the ‘introduced bird’ theory, believing that they were immigrants ‘from across the water’.”		
			Newsome and Noble 1986:17	Appendix 1 Approximate number of individuals released: “Self introduced.”	Sources not cited, and are unavailable where the paper says they are stored			
		Information from other literature:	Lever 1987:17	“In 1933, 20 Cattle Egrets were imported from India to Western Australia (6), where 18 were released along the Lennard River at Kimberley Downs Station near Derby. The birds soon disappeared, having probably fallen pretty to hawks, and there is no evidence that the present Australian population of Cattle Egrets are their descendants.”				

	12 – 21 (Chagos Archipelago)		Lever 1987:16<br><br>Lever cites Long 1981:71	“In 1955, a Captain Georges Lanier imported a dozen Cattle Egrets from the Seychelles to the Chagos Archipelago in an apparently successful attempt to control insect and other pests (it is possible that nine had previously been released in 1953), and by 1960 a colony of 27 nests was established at Point Est on Diego Garcia…”<br><br>“Twelve Cattle Egrets were deliberately introduced in 1955, from the Seychelles by Captain G. Lanier, in an attempt to control flies (Loustau-Lalanne 1962). Hutson (1975) says that according to the Seychellois in 1971, some nine Cattle Egrets were released in 1953.”	Hutson 1975:4	“According to the Seychellois resident in 1971, 9 cattle egrets were released in 1953 from the Seychelles. Loustau-Lalanne (1962) (who states that the species was introduced in 1955) found a colony of 27 nests at East point in 1960… the egret is now very common.	Uncertainty about the number and date of the introduction.	Loustau-Lalanne 1962:69, Bourne 1971:192

Laughing dove (Senegal turtledove) *Streptopelia senegalensis*	4 (Australia)	Duncan *et al.* 2001	Long 1981:222	"…introduced from the South Perth Zoological Gardens, Western Australia, from 1898 on (Serventy and Whittell 1951). They have spread rapidly since in the 1930s (Sedgewick 1957-59)...	Serventy and Whittell 1951:79	"Introduced from the South Perth Zoological Gardens since 1898…"	There is no reference to the specific number of birds introduced, as cited in Duncan *et al.* 2001. Moreover, the only number suggested is >>50 (Newsome and Noble 1986:18).	
					Sedgewick 1959:93	"[The Senegal turtledove was] 'introduced from South Perth Zoological Gardens since 1898' (Serventy and Whittell 1951)…(In a letter from Lt.-Col. E.A. Le Souef, April 27, 1936) I introduced them from the Melbourne Zoological Gardens, from memory about 1898 or 9…A few pairs have been sent to people in various parts of the country on request…"		
			Newsome and Noble 1986:18	Appendix 1 Approximate number of individuals released: "?>>50"	Sources not cited, and are unavailable where the paper says they are stored			
Laughing kookaburra	21 (New Zealand)	Veltman *et al* 1996					None of the sources cited by Veltman et al. specifies the number of birds introduced.	Parry 1973; Frith 1977:319; Blakers *et al.* 1984:325
Dacelo novaeguineae		Information from other literature:	Lever 1987:298	"In 1866 and 1869, the Otago Society liberated four and two, respectively,	Oliver 1930:435	"Several small shipments of the Laughing Kookaburra, or		

				near the Silverstream, where they remained for a time but eventually disappeared. In 1867, the Nelson Society imported some and a year later the Auckland Society received one from a Dr. Stratford – but once more their fate is unrecorded. In 1876 and 1870, 14 and 1 Laughing Kookaburras, respectively, were released by the Wellington Society; one was seen as late as 1885 but apparently none thereafter.<br><br>In the early 1860s, Sire George Grey released some Laughing Kookaburras on Kawau Island in Hauraki Gulf, Central Auckland, where according to Thomson they all died. In 1916, however, Thomson was told that a few		Laughing Jackass as it is often called, were liberated between 1866 and 1880, but none survived except those placed on Kawau Island by Sir George Grey. These gradually increased and eventually crossed to the mainland where they have been seen from the coast opposite Kawau to Devonport.”		

				were still to be found on the east coast near Auckland, and Oliver (1930 and 1955) indicates that Sir George Grey's introduction was the only successful one in New Zealand, and that from Kawau some crossed to the nearby mainland coast where they were common in the late 1920s…"				
Australasian magpie *Gymnorhina tibicen*	2 (Australia)	Duncan *et al.* 2001			Drummond 1906:243	"Many years ago a pair of these birds came over to Streamlands, in the Rodney County, from the Island of Kawau, when it was owned by Sir George Grey. They nested in a kauri-tree about a hundred yards from a settler's house, and from that spot they completely spread throughout the whole country."	Unconvincing data	

	10 (Auckland)	Duncan 1997	Long 1981:342	“The Auckland society introduced ten in 1867 and a single bird in 1870 (Thomson 1922).”	Thomson 1922:151	“The Auckland Society introduced ten in 1867, and one in 1870. But Sir George Grey introduced a number into Kawau probably at an earlier date; they very quickly become numerous, and spread to the mainland.”	Birds may have been introduced at an earlier date (Thomson 1922:150).<br><br>Multiple additional releases to nearby locations in the 1860-1870s, including Canterbury (68 ind., 1864-1871, Thomson 1922:150, Anderson 1916:21; 7 ind., 1867, Williams 1969:450; and <109 ind. in 1870, Thomson 1922:150, Lamb 1964:70), Otago (81 ind., 1865-1869, Thomson 1922:150), Wellington (260 ind., 1874, Thomson 1922:150), and Hawke’s Bay (unknown number, 1877, Wellwood 1968:218).	Buller 1888

Song Thrush *Turdus philomelos*	8 (Wellington)	Duncan 1997	Long 1981: 319	"…and by the Wellington society which released eight in 1878 (Thomson 1922)."	Thomson 1922:142	"The Wellington Society introduced eight in 1878."	Multiple other releases to nearby locations during the 1860 – 1870s (Turbott 1990:202), including Auckland (115 ind., 1867-1868, Thomson 1922: 142), Canterbury (80+ ind., 1867-1875, Thomson 1922:142, Williams 1969:443), and Otago (145 ind., 1865-1871, Thomson 1922:142).	Buller 1888:xlvii, Anderson 1916, Lamb 1964, Wellwood 1968
Red-whiskered bulbul *Pynconotus jocosus*	8 (Australia)	Duncan *et al.* 2001	Long 1981:296	"The Red-whiskered Bulbul appears to have been first introduced by the Zoological and Acclimatisation Society in 1880 in New South Wales (Tarr 1950). Some were reported at Homebush in 1902 and they appear to have been well established around Sydney in 1919-20.<br><br>Red-whiskered Bulbuls have turned up in Melbourne and Geelong in Victoria,	Tarr 1950:192	"Otocompsa emeria. Red-whiskered Bulbul. There is a record of bulbuls beings introduced by the Zoological Society in 1880, Bulbuls were also reported at Homebush in 1902."	There is no reference to the specific number of birds introduced, as cited in Duncan *et al.* 2001. The only suggestion is that birds may have been escapees, which are assumed to be small numbers (Newsome and Noble 1986:19).	MacPherson 1921, Wolstenholme 1921:74, Chaffer 1933:136

				but it does not appear to be known when they were liberated. In 1915-16 some were reported from Ashfield, and a pair were observed in Melbourne (Tarr 1950) in 1948.”				
		Information from other literature:	Lever 1987:313	“Red-whiskered Bulbuls (presumably of the nominate form) imported from China by the New South Wales Zoological Society in 1880… apparently disappeared. Others introduced about the turn of the century were more successful, and deliberately released and/or escaped birds became established around Sydney. Some were reported at Homebush in 1902 and at Double Bay in 1917…”				
	10-20 (USA)		Lever 1987:311	“In late July or early August 1960, a number of these birds (believed to have been between 5 and 10 pairs) that had been imported from Calcutta in eastern India… escaped from Alton Freeman’s Miami Rare Birds	Fisk 1966:10	“In late July or early August of 1960 – the exact date is unknown – a few birds escaped while being transferred from one aviary to another. There could not have been many, as Mr. Freeman was unaware of any loss	Actual number of birds that escaped is unreported, and appears to be based on a rough population model rather than data (Carleton and Owre 1975:43).	

				Farm in the suburb of Kendall in Dade County…”		until reports came in from his neighbors of a new bird…”		
		Information from other literature			Carleton and Owre 1975:43	“To produce the present population, a founding population of fewer than five feeding pairs would have had an annual rate of increase of more than 50% - which seems excessive. An initial population of more than 10 pairs seems to large from accounts of residents… Thus, from tentative data, we hypothesize that between 5 and 10 breeding pairs founding the population…”		

Eurasian tree sparrow *Passer montanus*	20-24 (USA)		Lever 1987:463	"On 20 April 1870, a bird-dealer named Kleinschmidt and Mr. carl Daenzer… released between 20 and 32 Tree Sparrows (which had been imported from Germany and were therefore of the nominate form) in Layfayette Park in suburban residential southern St. Louis, Missouri."	Phillips 1928:58	"According to a letter dated February 4, 1888, from C. Daenzer, and editor of the Anzeiger des Westerns, a German-language daily of St. Louis, Mo., 12 pairs of European tree sparrows were set free on April 25, 1870, in Lafayette park in that city… This seems to be the origin of the colony of this sparrow that has persisted in a small way in the vicinity of St. Louis ever since, but which was early driven out of the city by the stronger house sparrow, which occupied most of the available nesting sites. It is difficult to say just what the status of the bird in Missouri is today."		
					Cooke and Knappen 1941:182	"The European tree sparrow, a congener of the English sparrow, apparently was never liberated at any other place than St. Louis, Mo.,		

						where twelve pairs were freed in Lafayette Park in April, 1970… It has not spread far; in fact, it has never been observed more than a hundred miles from St. Louis in the seventy years since it was liberated.”		
					Wetmore 1964:291	“…was imported from Germany in 1870. A score of these brown-topped weaver finches were liberated in St. Louis, Missouri, park and the birds have barely spread beyond the city since.”		
					Barlow 1973:10	“Twenty individuals of *P. montanus* from Germany were released by Carl Daenzer in Lafayette Park, St. Louis, in late April of 1970.”		

House sparrow *Passer domesticus*	14 (Otago)	Duncan 1997	Long 1981:347	"In 1868-69 the Otago society liberated twenty-three sparrows (Thomson 1922)."	Thomson 1922:164	"The Otago Society liberated three in 1868, and 11 in 1869."	Two releases in Otago.<br><br>Multiple other releases to nearby locations in the 1860-1870s (Turbott 1990:221), including Auckland (59 ind., 1864-1867, Thomson 1922:164), Oliver 1930:511), Canterbury (95 ind., 1864-1868, Thomson 1922:164, Lamb 1964:66-69, Williams 1969:448), Nelson Society (7 ind., 1862-1871, Thomson 1922:164, Oliver 1930:511), Wanganui (~200 ind., 1865 or 1866, Buller 1888:xliv, Oliver 1930:511).	Anderson 1916:22-24, Wellwood 1968:218; Oliver 1930:511

Scaly-breasted munia (spice finch) *Lonchura punctulata*	8 (Australia)	Duncan *et al.* 2001	Long 1981:428	"…was first noted in Brisbane, Queensland, in about 1937. Lavery (1974) indicates the species probably escaped there in about 1930. They failed to gain much attention until the 1950s. Spice Finches were noted in Townsville in 1951 (Bell 1961; Lavery and Hopkins 1963) after liberation in 1950 (Lavery) and at Innisfail in 1955, Esk in 1955-56 and Mackay in 1959-60 (Bell). Some were reported from 193 km (120 miles) north of Cairns at Cooktown in 1961 and some were observed at Airlie and Noosaville in the same year (Wheeler 1962). They were first recorded at the Atherton Railway Station in June 1964 and are now well established and breeding there (Bravery 1970). The Spice Finch was	Bell 1961:96	"There can be no reasonable doubt that the Spice Finches now occurring in various parts of eastern coastal Australia, from Sydney to Cairns, originated from escapees from aviaries, or from birds purposely liberated. Years ago large numbers of these birds were brought into Australia on ships from south-east Asia and sold as cage birds."	There is no reference to the specific number of birds introduced at either the 1930 liberation or the 1950 escape, as cited in Duncan *et al.* 2001. The only suggestion is that birds may have been escapees, which are assumed to be small numbers (Newsome and Noble 1986:20).	Wheeler 1962, Lavery and Hopkins 1963:252, Bravery 1970:62, Lavery 1974:252
					McGill 1960:63	"Established from aviary escapes near Sydney about 1950, now well dispersed at Cumberland Plain. In north coastal regions, first recorded near Grafton about 1960, Taree in 1962, Lismore about 1969, Casino about 1972, and Murwillumbah in 1979. Also recorded near Moruya in 1973 and Cooyal in 1979."		

				also introduced into New South Wales (McGill 1960), also becoming established in that State as a result of escapes from aviaries. Although they were increasing their range and numbers there in the 1960s they had not spread far beyond the County of Cumberland boundaries (McGill 1960)."				
			Newsome and Noble 1985: 20	Appendix 1 Approximate number of individuals released: "Escapee"<br><br>Note: "Escapees are assumed to have been in small numbers" (2).	Sources not cited, and are unavailable where the paper says they are stored			

Chaffinch *Fringilla coelebs*	16 (Canterbury)	Duncan 1997	Long 1981:439	"The Canterbury society liberated some in 1867 (11), 1865 (5), and in 1871 (?), and three years later they were considered to be thoroughly established there…According to Thomson (1922) many more were released by private individuals and dealers at most of the principal centres of that time."	Thomson 1922:170	"The Canterbury Society liberated 11 in 1867, and five in 1868; and three years later reported that they are considered to be 'thoroughly established and to need no further importations.' In 1871 a further lot were introduced."	Multiple other releases in nearby locations during the 1860-1870s (Turbott 1990:218), including Auckland (113+ ind., 1864-1869), Otago (99 ind., 1868-1871), Wellington (126+ ind., 1874-1879), and private individuals (Thomson 1922:170).	Anderson 1916, Buller 1888:xlvii, Wellwood 1968
			Lamb 1964: 45	"The voyage had taken 94 days and the birds landed alive at Lyttelton were as follows: …11 chaffinches…The small birds were sold on condition of their 'being turned loose at once' (*Press* 18 F 1867, *Press* 19 F 1867)."	Unavailable		Duncan 1997 ignored listed introductions that did not report specific numbers. These unknown numbers could have influenced original populations.	
			Williams 1969:446	"In the Society's 1867 Annual Report eleven were reported to be kept in the Gardens and all of these were liberated in that year, plus another five in 1868, according to Thomson; though that this was indeed the	Thomson 1922:170	Same citation as listed above (Thomson 1922:170)		

				fate of all is not clear from the Annual Reports. However by 1869 flocks were continuously being seen in the Gardens and in 1870 the species was regarded by the Society as thoroughly established and in no need of further importations. Nevertheless, Thomson records another introduction in 1871. Thenceforward their occupation of Canterbury apparently progressed steadily.”				

Island canary *Serinus canaria*	12 (Midway Atoll, Hawaii))		Lever 1987:378	"In February and April 1910, 10 young hatched in [Mr. Daniel] Morrison's aviary and these, together with a further two males that arrived from Honolulu in July, were released later in the same year. (Lever notes that one account says 14 birds were released whereas another says only 11)."	Bailey 1956	Not in bibliography		Fisher and Baldwin 1945;13-14, Peterson 1961:276, Blake 1975:925, Pyle 1977:121, American Ornithologists Union 1983:669
					Berger 1981:220	"Bryan (1912) chronicled the establishment of the Canary on Midway Atoll. A pair was taken from Honolulu to Midway in March 1909; they were put into a breeding cage in January 1910. Ten young birds were raised, and these, together with 2 additional males from Honolulu, were released in July 1910. These birds began nesting in December the same year. It was estimated that about 60 young Canaries were raised in the wild during the first breeding season."		
					Bryan 1912:341	"In July [1910] two male canaries were sent me from Honolulu and these along with the eleven young birds which we had raised here were liberated."		

					Munro 1960: 175	"Many canaries escape from cages but from generations of care and confinement they are not adapted to care for themselves and guard against enemies in the open except in rare cases. One such case is Sand Island of the Midway Islands… The Morrison brought several lots of tame canaries to the island and bred them there. They released eleven."		

European Greenfinch *Carduelis chloris*	8 (Otago)	Duncan 1997	Long 1981:448	"…and the Otago Society liberated eight in 1868…(Thomson 1922).	Thomson 1922:174	"The Otago Society liberated eight in 1868."	Multiple other releases to nearby locations in the 1860s (Turbott 1990:219), including Auckland, (51+ ind., 1865-1868, Thomson 1922:174), Nelson (5 ind., 1862, Thomson 1922:174), and Hawke's Bay (Wellwood 1968:218). Birds may have been introduced to Canterbury, but alternative use of common names (green linnet, linnet) have made these introductions difficult to pinpoint (Williams 1969:444).	Buller 1888:xlvii, Anderson 1916:22

Common redpoll *Carduelis flammea*	2 (Wellington)	Duncan 1997	Long 1981:454	"…The Wellington society also introduced two birds in the same year [1875] (Thomson 1922)."	Thomson 1922:172	"The Wellington Society introduced two in 1875."	Multiple other releases to nearby locations between 1862 and 1875 (Turbott 1990:220), including Auckland (210 ind., 1871-1872), Canterbury (134+ ind., 1869-1875), and Otago (81 ind., 1868-1871), each instance being reported by Thomson 1922:172.<br><br>There is uncertainty about the exact species of redpoll that was introduced to New Zealand (Williams 1969:446).	Buller 1888:xlvii, Anderson 1916:22, Lamb 1964:45, Wellwood 1968
Cirl bunting *Emberiza cirlus*	Multiple authors reported the same suite of introductions differently. The suite is described to the right (Long 1981:466), and the different reports are shown below		Long 1981:466	"The Otago Acclimatisation Society liberated seven birds in 1871 (Thomson 1922) and four birds were liberated at Wellington in 1880 (Oliver 1930). Some eighteen birds were also liberated on Stewart Island in 1879, but the species apparently failed to become established on that island."	Thomson 1922:175	"The Otago Society liberated seven in 1981. They seem at once to have increased and spread… In 1879 18 were liberated in Stewart Island, but they failed to establish themselves. Their occurrence is very erratic. At one time they increased to a very considerable extent in Otago;	Multiple releases in close proximity, including Otago (7 ind., 1880, Thomson 1922) and Wellington (4 ind., 1881, Oliver 1930: 514).<br><br>Turbott 1990:218 suggests that other introductions may have been made, but not recorded.<br><br>Lever 1987:353 suggests that the Cirl bunting could have	

					then they seemed to be quite rare. Now they are more common again…If all the cirl buntings now in New Zealand are descended from the seven originally liberated in Otago, the case is certainly a very interesting one.”	been mistaken for Yellowhammers (*Emberiza citronella*).	
				Oliver 1930:514	“The only records of the introduction of the Cirl Bunting to New Zealand given by the Hon. G.M. Thomson, are seven birds liberated in Otago in 1871 and four in Wellington in 1880. Evidently both lots became established as the species is now found in several localities in both islands. In 1879, eighteen birds were transferred to Stewart Island.”		

	29 (New Zealand)	Veltman *et al* 1996	Long 1981:466	Long reported all introductions as a single introduction	Thomson 1922:175	Reports 7 birds successfully introduced to New Zealand (Otago).		Groh 1975;13-14, Barba and Lopez 1990
					Oliver 1930:514	Reports 11 (Otago + Wellington) birds successfully introduced to New Zealand.		
	7 (Otago, NZ)	Duncan 1997	Long 1981:466	The 1871 release (Thomson 1922)	Thomson 1922:175	Reports 7 birds successfully introduced to Otago.		Buller 1888, Anderson 1916, Lamb 1964, Wellwood 1968, Williams 1969
					Turbott 1990:218	“Only two small introductions have been documented (7 birds to Otago in 1871 and 4 to Wellington in 1880-81), but probably others were made and not recorded.”		
	4 (Wellington, NZ)	Duncan 1997	Long 1981:466	The 1880 release (Oliver 1930)	Oliver 1930:514	Reports 4 successfully introduced to Wellington.		Buller 1888, Anderson 1916, Lamb 1964, Wellwood 1968, Williams 1969
					Turbott 1990:218	Reported above.		
		Information from other literature:	Lever 1987:353	“Thomson once discovered several Cirl Buntings in a cage allegedly containing only Yellowhammers (*Emberiza citronella*) – the				

				females and young of which quite closely resemble those of *E. cirlus* – it seems probable that others may have been unwittingly imported.			

REFERENCES

American Ornithologists' Union. 1983 *Check-list of North American birds.* 6th edition. Lawrence, Kansas.

Anderson, J.C. 1916 *Jubilee history of South Canterbury*. Whitcomb & Tombs, Auckland.

Balmford, R. 1978 Early introductions of birds to Victoria. *Aust. Bird Watcher* 7, 237-248.

Barba, E., & Lopez, J.A. 1990 Breeding season, clutch size, and breeding success in the Cirl Bunting *Emberiza cirlus.* Mediterranea Serie de Estudios Biologicos 12, 79-88.

Barlow, J.C. 1973 Status of the North American population of the European Tree Sparrow. In *Symposium on the House Sparrow and European Tree Sparrow in North America* (ed S.C. Kendeigh), pp. 10-23. Ornithological Monograph No. 14. Lawrence, Kansas: American Ornithologist' Union.

Bell, H.L. 1961 The introduced spice finch in north-eastern Queensland. *Emu* 61, 94-96.

Berger, A.J. 1981 *Hawaiian birdlife*. Honolulu, Hawaii: University Press.

Blake, C.H. 1975 Introductions, transplants, and invaders. *American Birds* 29, 923-926.

Blakers, M., Davies, S.J.J.F., & Reilly, P.N. 1984 *The atlas of Australian birds*. Melbourne: Melbourne University Press

Bourne, W.R.P. 1971 The birds of the Chargos Group, Indian Ocean. *Atoll Research Bulletin* 149, 175-207.

Bravery, J.A. 1970 Birds of the Atherton Shire, Queensland. *Emu* 70, 49-63.

Bryan, W.A. 1912 The introduction and acclimatization of the Yellow Canary on Midway Island. *Auk* 29, 339-340.

Buller, W.L. 1888 *A History of the birds of New Zealand.* 2d ed. London.

Carleton, A. R., & Owre, O.T. 1975 The red-whiskered bulbul in Florida: 1960-71. *Auk* 92, 40-57.

Chaffer, N. 1933 The Bulbul. *Emu* 33, 136-137.

Chalmers, C.E. 1972 Cattle Egret in the Gippsland Area. *Emu* 72, 180-181.

Cooke, M.T. & Knappen, P. 1941 Some birds naturalized in North America. *Trans. 5th North American Wildlife Conf.*, 176-183.

Cramp, S. 1977 *Handbook of the birds of Europe, the Middle Easy, and North Africa*. Vol 1. Oxford: Oxford University Press.

Drummond, J. 1906 On introduced birds. *Trans. Proc. New Zealand Inst.* 39, 227-252.

Duncan, R. P. 1997 The role of competition and introduction effort in the success of passeriform birds introduced to New Zealand. *Amer. Nat.* 149, 903-915.

Duncan, R.P., Bomford, M., Forsyth, D.M., & Conibear, L. 2001 High predictability in introduction outcomes and the geographical range size of introduced Australian birds: a role for climate. *J. Anim. Ecol.* 70, 621-632.

Falla, R.A., Sibson, R.B., & Turbott, E.G. 1966 *A field guide to the birds of New Zealand*. Collings, London

Fisher, H.I. & Baldwin, P.H. 1945 A recent trip to Midway Islands, Pacific Ocean. *Elepaio* 6, 11-13.

Fisk, E.J. 1966 A happy newcomer in a fruitful land. *Florida Naturalist* 39, 10-11.

Frith, H.J. 1977 *Reader's Digest complete book of Australian birds*. Sydney: Reader's Digest Services.

Groh, G. 1975 Zür Biologie der Zaunammer (*Emberiza cirlus* L.) in der Pfalz. *Mitteilungen der Pollichia* 63, 72-139.

Gruiler, E.R. 1967 The Cape Barren goose, its environment, numbers and breeding. *Emu* 66, 211-235.

Hewitt, J.M. 1960 The Cattle Egret in Australia. *Emu* 60, 99-102.

Hutson, A.M. 1975 Observations on the birds of Diego Garcia, Chargos Archipelago, with notes on other vertebrates. *Atoll Res. Bull.* 75, 1-25.

Jenkins, C.F.H. 1977 The Noah's Ark Syndrome, Perth, Western Australia: General Printing

Jenkins, C.F.H. & Ford, J. 1960 The cattle egret and its symbionts in South-Western Australia. *Emu* 60, 245-249.

Lamb, R.C. 1964 *Birds, beasts and fishes*. Caxton, Christchurch.

Lavery, H.J. 1974 Species introductions by man. In: *Fauna of Queensland Yearbook 8-9*, Brisbane: Government Printers

Lavery, H.J. &Hopkins, N. 1963 Birds of the Townsville District of North Queensland. *Emu* 63, 242-252.

Lever, C. 1987 *Naturalized birds of the world*. New York: Longman Sci. Tech.

Long, J.L. 1981 *Introduced birds of the world*. New York: Universe.

Loustau-Lalanne, P. 1962. The birds of the Cagos Archipelago, Indian Ocean. Ibis 104:67-73.

MacPherson, J. 1921 The red-eyed bulbul. *Emu* 23, 218-219.

Marriott, R.W., & D.K. Forbes. 1970 The digestion of Lucerne chaff by Cape Barren geese, Cereopsis novaehollandiae Latham. *Australian J. Zool.* 18, 257-263.

McGill, A.R. 1960 A handlist of the birds of New South Wales. Sydney: Fauna Protection Panel.

Morgan, B. & Morgan, J. 1965 Cattle egrets in Brisbane. *Emu* 64, 230-232.

Munro, G.C. 1960 *Birds of Hawaii*. Japan: Bridgeway Press.

Newsome, A.E & Noble, I.R. 1986 Ecological and physiological characters of invading species. In: *Ecology of Biological Invasions* (eds R.H. Groves & J.J. Burdon), pp.1-20. Cambridge: Cambridge University Press.

Oliver, W.R.B. 1930 *Birds of New Zealand*. Wellington, New Zealand: Fine Arts.

Oliver, W.R.B. 1955 *Birds of New Zealand, 2nd ed.* Wellington, New Zealand: Reed.

Parry, V. 1973 The auxiliary social system and its effect on territory and breeding in kookaburras. *Emu* 73, 81-100.

Peterson, R.T. 1961 A *Field Guide to Western Birds*. Boston, MA: Houghton Mifflin.

Phillips, J.C. 1928 Wild birds introduced or transplanted in North America. *Technical Bull. No. 61*. Washington D.C.

Pyle, R.L. 1977 Preliminary list of the birds of Hawaii. *Elepaio* 37, 110-121.

Sedgewick, E.H. 1958 The introduced turtledoves in Western Australia. *West Aust. Nat*. 6, 92-127.

Serventy, D.L. & Whittell, H.M. 1948 A *systematic list of the birds of Western Australia*. Perth, Western Australia: Govt. Printer.

Serventy, D.L. & Whittell, H.M. 1967 *Handbook of the birds of Western Australia*, 4th edn. Perth: Paterson Brokensha

Tarr, H.E. 1950 The distribution of foreign birds in Australia. *Emu* 49, 189-195.

Thomson, G.M. 1922 The *naturalization of animals and plants in New Zealand*. London: Cambridge University Press.

Turbott, E.G. 1990 *Checklist of the birds of New Zealand and the Ross Dependency, Antarctica*. Auckland: Random Century.

Veltman, C. J., Nee, S., & Crawley, M. J. 1996 Correlates of introduction success in exotic New Zealand birds. *Amer. Nat.* 147, 542-557.

Wellwood, J.M. 1968 *Hawke's Bay Acclimitization Society centenary 1868-1968*. Hastings: Hawke's Bay Acclimatization Society

Wetmore, A. 1964 *Song and garden birds of North America*. Washington, D.C: National Geographic Society.

Wheeler, J.R. 1962 Observations on the Cattle Egret in Eastern Australia. *Emu* 62, 192-93.

Williams, G.R.1968 The cape barren goose (*Cereopsis novaehollandiae* Latham) in New Zealand. *Notornis* 15, 66-69.

Williams, G.R. 1969 Introduced birds. In *The natural history of Canterbury*. (ed G.A. Knox), pp. 435-451. Reed, Wellington.

Wodzicki, K. 1965 The status of some exotic vertebrates in the ecology of New Zealand. In: *Genetics of colonizing species*. (eds H.G. Baker and A.L. Stebbins), Proc. 1st int. Unl. Biol. Sci., Symp. Gen. Biol., New York and London: Academic Press.

Wolstenholme, H. 1921 Letter regarding the Red-eyed Bulbul. *Emu* 21, 74.
